# Supplementary material for: Infection with intestinal helminth (Hymenolepis diminuta) impacts exploratory behavior and cognitive processes in rats by changing the central level of neurotransmitters
Source: PLoS Pathog. 2022 Mar 14;18(3):e1010330. doi: 10.1371/journal.ppat.1010330 (PMC8947016; doi:10.1371/journal.ppat.1010330)

PLOS Pathogens

Infection with intestinal helminth (Hymenolepis diminuta) impacts exploratory behavior and cognitive processes in rats by decreasing noradrenergic and serotonergic neurotransmission.

--Manuscript Draft--

**Manuscript Number:**	PPATHOGENS-D-21-02092
**Full Title:**	Infection with intestinal helminth (Hymenolepis diminuta) impacts exploratory behavior and cognitive processes in rats by decreasing noradrenergic and serotonergic neurotransmission.
**Short Title:**	Tapeworm infection impacts rat behavior and neurotransmission
**Article Type:**	Research Article
**Section/Category:**	Parasitology
**Keywords:**	behavior, CNS, Hymenolepis diminuta, neurotransmission, parasites, tapeworm infection
**Abstract:**	Parasites may significantly affect the functioning of the host organism, with some even being able to invade the brain and alter the host behavior. The impact of intestinal worms on the host central nervous system (CNS) remains unexplored. The aim of this study was to evaluate the effect of intestinal infection by the tapeworm Hymenolepis diminuta on behavior and functions of the CNS in rats. The 3 months old animals were infected, and the effects on anxiety, exploration, sensorimotor skills and learning processes were assessed at 18 months in Open Field, Novel Object Recognition and the Water Maze tests. After completing the behavioral studies, both infected and non-infected rats were sacrificed, and the collected tissues were subjected to biochemical analysis. The levels of neurotransmitters, their metabolites and amino acids in selected structures of the CNS were determined by HPLC. In addition, the gene expression profile of the pro- and anti-inflammatory cytokines (TNF-α, IL-1β, IL-6 and IL-10) was evaluated by Real-Time PCR to determine the immune response of the CNS to the tapeworm infection. The parasites caused significant changes in exploratory behavior, most notably, a reduction of velocity and total distance moved in the OF test; the infected rats exhibited decreased frequency in the central zone, which may indicate a higher level of anxiety. Additionally, parasite infestation improved spatial memory, assessed in the WM test, and recognition of new object s. These changes are related to the identified reduction in noradrenaline level in the CNS structures and less pronounced changes in striatal serotonergic neurotransmission. H. diminuta infestation was also found to cause a significant reduction of hippocampal expression of IL-6. Our results provide new data for further research on brain function during parasitic infections especially in relation to helminths and diseases in which noradrenergic system may play an important role.
**Additional Information:**	
**Question**	**Response**
**Financial Disclosure**<br><br>Enter a financial disclosure statement that describes the sources of funding for the work included in this submission. Review the submission guidelines for detailed requirements. View published research articles from *PLOS Pathogens* for specific examples.<br><br>This statement is required for submission and **will appear in the published article** if the submission is accepted. Please make	This research was partially supported by the National-Science-Center-Poland (Grant Number 2014/13/B/NZ6/00881).<br>Project implemented with CePT infrastructure financed by the European Union - the European Regional Development Fund within the Operational Programme "Innovative economy" for 2007-2013".

Powered by Editorial Manager® and ProduXion Manager® from Aries Systems Corporation

sure it is accurate.<br><br>**Unfunded studies**<br>Enter: *The author(s) received no specific funding for this work.*<br><br>**Funded studies**<br><br>Enter a statement with the following details:<br>• Initials of the authors who received each award<br>• Grant numbers awarded to each author<br>• The full name of each funder<br>• URL of each funder website<br>• Did the sponsors or funders play any role in the study design, data collection and analysis, decision to publish, or preparation of the manuscript?<br>• **NO** - Include this sentence at the end of your statement: *The funders had no role in study design, data collection and analysis, decision to publish, or preparation of the manuscript.*<br>• **YES** - Specify the role(s) played.<br><br>* typeset	
**Competing Interests**<br><br>Use the instructions below to enter a competing interest statement for this submission. On behalf of all authors, disclose any competing interests that could be perceived to bias this work—acknowledging all financial support and any other relevant financial or non-financial competing interests.<br><br>This statement **will appear in the published article** if the submission is accepted. Please make sure it is accurate. View published research articles from *PLOS Pathogens* for specific examples.	The authors have declared that no competing interests exist.

**NO authors have competing interests**<br><br>Enter: *The authors have declared that no competing interests exist.*<br><br>**Authors with competing interests**<br><br>Enter competing interest details beginning with this statement:<br><br>*I have read the journal's policy and the authors of this manuscript have the following competing interests: [insert competing interests here]*<br><br>* typeset<br><br>This statement is required for submission and **will appear in the published article** if the submission is accepted. Please make sure it is accurate and that any funding sources listed in your Funding Information later in the submission form are also declared in your Financial Disclosure statement.	
**Data Availability**<br><br>Authors are required to make all data underlying the findings described fully available, without restriction, and from the time of publication. PLOS allows rare exceptions to address legal and ethical concerns. See the PLOS Data Policy and FAQ for detailed information.<br><br>A Data Availability Statement describing where the data can be found is required at submission. Your answers to this question constitute the Data Availability Statement and **will be published in the article**, if accepted.<br><br>**Important:** Stating 'data available on request from the author' is not sufficient. If your data are only available upon request, select 'No' for the first question and explain your exceptional situation in the text box.	Yes - all data are fully available without restriction

Do the authors confirm that all data underlying the findings described in their manuscript are fully available without restriction?	
**Describe where the data may be found in full sentences. If you are copying our sample text, replace any instances of XXX with the appropriate details.**<br>• If the data are **held or will be held in a public repository**, include URLs, accession numbers or DOIs. If this information will only be available after acceptance, indicate this by ticking the box below. For example: *All XXX files are available from the XXX database (accession number(s) XXX, XXX.*).<br>• For research data submitted to Dryad, enter the following: *The data is available from Dryad repository DOI: XXX*.<br>• If the data are all contained **within the manuscript and/or Supporting Information files**, enter the following: *All relevant data are within the manuscript and its Supporting Information files.*<br>• If neither of these applies but you are able to provide **details of access elsewhere**, with or without limitations, please do so. For example:<br><br>*Data cannot be shared publicly because of [XXX]. Data are available from the XXX Institutional Data Access / Ethics Committee (contact via XXX) for researchers who meet the criteria for access to confidential data.*<br><br>*The data underlying the results presented in the study are available from (include the name of the third party and contact information or URL).*<br>• This text is appropriate if the data are owned by a third party and authors do not have permission to share the data.<br><br>* typeset	ll relevant data are within the manuscript and its Supporting Information files.
Additional data availability information:	

Powered by Editorial Manager® and ProduXion Manager® from Aries Systems Corporation

Manuscript

Click here to access/download;Manuscript;Hymenolepis -CNS PLos 15.10.2021.docx

Tapeworm infection impacts rat behavior and neurotransmission

INFECTION WITH INTESTINAL HELMINTH (HYMENOLEPIS DIMINUTA) IMPACTS EXPLORATORY BEHAVIOR AND COGNITIVE PROCESSES IN RATS BY DECREASING NORADRENERGIC AND SEROTONERGIC NEUROTRANSMISSION.

Kamilla Blecharz-Klin[1], Magdalena Świerczyńska[1], Agnieszka Piechal[1], Adriana Wawer[1], Ilona Joniec-Maciejak[1], Justyna Pyrzanowska[1], Ewa Wojnar[1], Anna Zawistowska-Deniziak[3], Anna Sulima-Celińska[2], Daniel Młocicki [2,3*], Dagmara Mirowska-Guzel[1*]

[1]***Department of Experimental and Clinical Pharmacology,***
Medical University of Warsaw, Centre for Preclinical Research and Technology CePT
Banacha 1B, 02-097 Warsaw, Poland
[2]***Department of General Biology and Parasitology, Medical University of Warsaw,***
Chałubińskiego 5, 02-004 Warsaw, Poland
[3]***W. Stefański Institute of Parasitology, Polish Academy of Sciences, Twarda 51/55 00-818 Warsaw, Poland***

[*] Corresponding Authors:
Dagmara Mirowska-Guzel
Department of Experimental and Clinical Pharmacology,
Medical University of Warsaw, Centre for Preclinical Research and Technology CePT
Banacha 1B, 02-097 Warsaw, Poland

tel./fax. +48 22 116 6202
E-mail address: dagmara.mirowska-guzel@wum.edu.pl

Daniel Młocicki
Department of General Biology and Parasitology, Medical University of Warsaw, Chałubińskiego 5, 02-004 Warsaw, Poland
tel./fax. +48 22 621 26 07
E-mail address: daniel.mlocicki@wum.edu.pl

ABSTRACT

Parasites may significantly affect the functioning of the host organism, with some even being able to invade the brain and alter the host behavior. The impact of intestinal worms on the host central nervous system (CNS) remains unexplored. The aim of this study was to evaluate the effect of intestinal infection by the tapeworm *Hymenolepis diminuta* on behavior and functions of the CNS in rats. The 3 months old animals were infected, and the effects on anxiety, exploration, sensorimotor skills and learning processes were assessed at 18 months in Open Field, Novel Object Recognition and the Water Maze tests. After completing the behavioral studies, both infected and non-infected rats were sacrificed, and the collected tissues were subjected to biochemical analysis. The levels of neurotransmitters, their metabolites and amino acids in selected structures of the CNS were determined by HPLC. In addition, the gene expression profile of the pro- and anti-inflammatory cytokines (TNF-α, IL-1β, IL-6 and IL-10) was evaluated by Real-Time PCR to determine the immune response of the CNS to the tapeworm infection. The parasites caused significant changes in exploratory behavior, most notably, a reduction of velocity and total distance moved in the OF test; the infected rats exhibited decreased frequency in the central zone, which may indicate a higher

level of anxiety. Additionally, parasite infestation improved spatial memory, assessed in the WM test, and recognition of new objects. These changes are related to the identified reduction in noradrenaline level in the CNS structures and less pronounced changes in striatal serotonergic neurotransmission. *H. diminuta* infestation was also found to cause a significant reduction of hippocampal expression of IL-6. Our results provide new data for further research on brain function during parasitic infections especially in relation to helminths and diseases in which noradrenergic system may play an important role.

Author Summary

Recent advances in the research on parasitic manipulation and/or control of the nervous system of their host resulted in the development of neuro-parasitology, a new and emerging branch of science. There have been advances in this area in relation to parasite-insect interactions or parasites directly invading central nervous system (CNS). However, the neuro-parasitology of parasitic infections in vertebrate hosts remains unexplored. In our study we evaluated the effect of intestinal infection by the tapeworm on the behavior, neurotransmission and functions of the CNS in rats. This infection positively influences spatial memory and new object recognition. At the same time, the infected animals develop a greater level of anxiety and move more slowly. These behavioral changes were related to the reduction in noradrenaline level in the CNS structures, and less pronounced changes in striatal serotonergic neurotransmission. These results provide important data for the further progress in neuro-parasitology and our understanding of parasite-host interactions. In our opinion in the near future may turn out that the role of the intestinal macrobiome in the CNS functioning may be just as significant as that of the microbiome.

Keywords: behavior, CNS, *Hymenolepis diminuta*, neurotransmission, parasites, tapeworm infection

1. Introduction

The small intestine is a hostile environment for parasitic worms including *Hymenolepis diminuta*, being subject to digestive enzymes, immune response components, bacteria and active peristaltic movements. To provide stability, adult parasites utilize their scolex and tegumental microtiches to anchor to the intestinal epithelium, thus allowing parasite-derived molecules like excretory-secretory, surface, and tegumental proteins to interact with the host immune system [1-2]. Many of these molecules are proteins involved in the parasite metabolism and survival strategies, and can play significant roles in the parasite-host relationship. The presence of adult tapeworms may influence the function of the host intestines, thereby affecting the intestinal processes, immunity and microbiome [3-5]. Studies into gut-brain crosstalk have revealed a complex communication system likely to have multiple effects on behavior and cognitive functions [6]. The complexity of these interactions is held within the *gut-brain axis* (GBA). Recent research has provided a new insight into the interactions taking place between enteric microbiota and the central and enteric nervous systems, indicating the causal relationship between gut microbiota and brain functions [7-9]. Therefore, it seems highly probable that also intestinal parasites, may influence their surrounding environment and, consequently, the brain function and behavior of the host.

Parasite invasion may have a serious effect on the host organism and activate immune responses. Such responses may result in phenotypic changes that could easily be interpreted as the results of parasite manipulation [10]. One example meriting particular note is invasion of the host brain, which can alter the host behavior in many ways; however, the response of the brain to these infections remains unclear. In most cases, such invasions improve parasite

dissemination by altering host behavior, with these effects being predominantly negative for the host. However, present study indicates that infection with the intestinal helminth *Hymenolepis diminuta* might have effects on the behavior of the definitive host and impact neurotransmission in the brain. It has been suggested that *H. diminuta* infection may offer therapeutic potential by protecting the host from autoimmune diseases. Furthermore, experiments on animal models indicate that *H. diminuta* ameliorates inflammatory diseases such as inflammatory bowel disease, and protect against chemically-induced colitis [5, 11]. Moreover, recent reports indicate that intestinal helminths, including *H. diminuta*, play important roles in modeling the composition of the intestinal microbiome and its impact on the functioning of the immune and nervous systems [12-14]. These studies suggest that early colonization by commensal organisms such as helminths causes modulation of the immune system, thus allowing them to positively influence the development of neuroinflammatory and cognitive disorders [13].

Since only very limited data are available on the effect of tapeworm infestation on behavior and brain function in a host, the aim of the present study was to evaluate the impact of *H. diminuta* tapeworm infection on rat behavior, with a particular emphasis on learning memory consolidation, anxiety, motor skills and the levels of selected neurotransmitters. It also examines the gene expression for pro- and anti-inflammatory cytokines in central nervous system (CNS) structures. It reveals new data on the impact of *H. diminuta* infection on cognitive processes and brain neurotransmission, and thus on the interactions between host and parasite. Our findings may serve as a valuable base for further research on the influence of parasitic infections on host brain function, and may indicate a new path in the study on neurodegenerative diseases characterized by noradrenergic system dysfunctions.

2. Results

2.1. Parasitic infection inhibited Open Field (OF) behavior

It was found that in male rats, parasitic infection inhibited Open Field behavior. Compared to the control group, infected rats demonstrated a shorter total path length in the test (Con: 13.52±0.004 m, Hym: 11.084±0.003 m; $F_{(1,13)}$=11.5762, p=0.004) (Fig. 1A), lower velocity of movement (Con: 0.07±0.0002, Hym: 0.06±0.0002 m/s; $F_{(1,13)}$=12.435, p=0.004) (Fig. 1B) and smaller frequency central part of the apparatus (Con: 12.00±0.63, Hym: 7.00±0.54; $F_{(1,13)}$=17.843, p=0.001) (Fig. 1C) as well as spent less time in the central area (Con: 43.215±3.18 s, Hym: 26.726±3.38 s; $F_{(1,13)}$=6.316, p=0.026) (Fig. 1D).

Infestation with *H. diminuta* also caused a reduction in grooming (Con: 2.375±0.24, Hym: 1.143±0.23; $F_{(1,13)}$=6.866, p=0.021). However, the infected rats did not demonstrate any significant change in climbing (Con: 8.625±1.61, Hym: 5.429±0.58; $F_{(1,13)}$=1.644, p=0.222), frequency of urination (Con: 0.750±0.12, Hym: 0.429±0.14; $F_{(1,13)}$=1.560, p=0.234) and defecation (Con: 2.375±0.58, Hym: 2.571±0.63; $F_{(1,13)}$=0.026, p=0.874) compared to the control.

2.2. Novel Object Recognition (NOR) is higher in infected rats

During the familiarization phase (day 1) of NOR, the infected rats spent more total time on exploring two identical objects (Con: 10.165±0.887 s, Hym: 23.223±2.522 s; $F_{(1,13)}$=4.755, p=0.0482) but their velocity was lower (Hym: 0.043±0.006 m/s) compared to the control group (Con: 0.064±0.006 m/s) ($F_{(1,13)}$=6.228, p=0.027).

H. diminuta infection had no effect on the other measured parameters, such as frequency of climbing on object A1 ($F_{(1,13)}$=1.053, p=0.323) and A2 ($F_{(1,13)}$=0.043, p=0.838), latency to first contact with any object ($F_{(1,13)}$ = 2.653, p=0.127), cumulative time exploring object A1 ($F_{(1,13)}$=3.101, p=0,102) and A2 ($F_{(1,13)}$=1.786, p=0.204).

During the choice phase (day 2) it was observed that the infected animals climbed the new object (B) significantly more often than the control rats (Con: 0.625±0.32, Hym: 2.143±0.40;

$F_{(1,13)}$=8.782, p=0.011). However, no statistically significant differences were observed in the velocity ($F_{(1,13)}$=3.329, p=0.091), total time of contact with both object (F=2.741, p=0.122), or the cumulative time exploring the old object ($F_{(1,13)}$=0.729, p=0.409) or the new object ($F_{(1,13)}$=0.800, p=0.387).

No differences in the value of Discrimination Index (DI) were found between experimental groups for the choice phase (Con: -0.292±1.212, Hym: -0.358±0.981; $F_{(1,13)}$=0.002, p=0.968). A negative DI score indicated that the animals spent more time with a familiar object.

Infestation with *H. diminuta* did not significantly influence the Index of Global Habituation (GHI) (Con: -0.684±0.350, Hym: -0.738±0.158; $F_{(1,13)}$=0.176, p=0.897) or the Recognition Index (RI) (Con: 2.294±1.039, Hym: 1.578±0.725; $F_{(1,13)}$=0.301, p=0.593).

2.3. Water maze (WM) results

2.3.1. Acquisition phase (days 1-4, trials 1-16)

No statistically significant differences in the latency associated with learning the platform location (trials 1-16) were found between the control group (Con: 30.227±7.866 s) and infected rats (Hym: 29.357±8.845 s) during the four day trial, as evaluated by ANOVA with repeated measurements ($F_{(1,13)}$=0.036, p=0.853).

Significant differences were, however, found between groups in the velocity of swimming on days 3 and 4 of learning ($F_{(1,13)}$= 9.488, p=0.003 and $F_{(1,13)}$=11.611, p=0.001 respectively). The rats infected with *H. diminuta* moved more slowly compared to the control animals (day 3: Con: 0.221±0.002 m/s, Hym: 0.188±0.001 m/s; day 4: Con: 0.223±0.001 m/s, Hym: 0.187±0.002 m/s). No differences in the speed of movement were noted on the remaining days, or in the distance travelled by the animals during the acquisition phase.

2.3.2. Probe trial - memory test I (day 5, trial 17)

The rats infected with *H. diminuta* demonstrated an insignificant increase in the number of crossings over the previous position of the platform during the memory test conducted on day

5 of the Water Maze (Con: 2.25±0.618, Hym: 3.29±0.746; $F_{(1,13)}$=1.159, p=0.301) (Fig. 2A). A more detailed analysis showed that infected animals spent more time in the close zone around the platform (Con: 1.065±0.312 s, Hym: 2.280±0.426 s; $F_{(1,13)}$=5.462, p=0.036) as well as in the SE quadrant (Con: 15.225±1.164 s, Hym: 23.211±2.355 s; $F_{(1,13)}$=9.972, p=0.007) One-way ANOVA found no difference between the experimental groups, either in distance moved (Con: 1.31±0.054 m, Hym: 1.22±0.042 m; $F_{(1,13)}$=1.772, p=0.206) or velocity (Con: 0.22±0.009 m/s, Hym: 0.21±0.007 m/s; $F_{(1,13)}$=1.772, p=0.206).

2.3.3. Repeated training (day 8, trials 18-21) indicates shorter latency to the hidden underwater platform in *H. diminuta*-infected rats

After a two-day break, training resumed. It was found that the infected group demonstrated significantly shorter latency to the hidden underwater platform (12.607±1.006 s) than the control group (20.625±0.880 s)($F_{(1,13)}$=4.820, p=0.047). No significant difference in swimming distance ($F_{(1,13)}$=0.002, p=0.968) or speed ($F_{(1,13)}$=0.049, p=0.829) was found on the same day.

2.3.4. Reversal platform (day 9, trials 22-25) – infected rats more frequently crossed the position of the old platform

On day 9 of the Water maze test, the hidden platform was translocated to the NW quadrant. ANOVA for repeated measures showed significant differences in frequency ($F_{(1,13)}$=4.842, p=0.032) and cumulative duration around old platform position ($F_{(1,13)}$=10.892, p=0.002). The rats with experimental hymenolepidosis crossed the position of the old platform more frequently then controls (Con: 0.219±0.173, Hym: 0.357±0.256) and spent more time around the previous position of the underwater platform (Con: 0.068±0.054, Hym: 0.120±0.097). No statistically significant differences were noted in latency to the new platform ($F_{(1,13)}$=0.001, p=0.981) or cumulative time spent around the new platform location ($F_{(1,13)}$=0.002, p=0.967). The swimming velocity was lower in the infected group than the control group ($F_{(1,13)}$=8.243,

p=0.006); however, no significant differences in swimming distance were observed between groups ($F_{(1,13)}$= 0.227, p=0.635).

2.3.5. Probe trial - memory test II (day 10, trial 26)

The memory test on day 10 revealed significant differences in the number of crossings over the original position of the platform in the SE quadrant between the groups (Con: 1.625±0.263, Hym: 2.857±0.260; $F_{(1,13)}$=10.945, p=0.006) (Fig. 2B). However, no difference was found between the treatment groups in the number of crossings over the new position of the platform in the NW sector according to one-way ANOVA (Con: 1.500±0.378, Hym: 2.571±0.428; $F_{(1,13)}$=3.545, p=0.08) (Fig. 2C) .

No significant difference was found between the experimental groups for distance moved (Con: 1.43±0.052 m, Hym: 1.31±0.049 m; $F_{(1,13)}$=2.471, p=0.140) or velocity (Con: 0.24±0.009 m/s, Hym: 0.22±0.008 m/s; $F_{(1,13)}$=2.471, p=0.140).

2.3.6. Visible platform test (day 11, trials 27-30)

On day 11, the dark-marked platform was raised above the water surface. No differences were found between the groups reading latency to reach the visible platform ($F_{(1,13)}$=0.099, p=0.961), distance moved ($F_{(1,13)}$= 0.596, p=0.622) or velocity ($F_{(1,13)}$=1.511, p=0.227).

2.4. HPLC indicates alteration in neurotransmitters of infected rats

The infected rats demonstrated significantly altered levels of several neurotransmitters in distinct brain regions compared with uninfected animals. The experimental hymenolepidosis in the rats was associated with a significant reduction of NA in the prefrontal cortex (Con: 148.34±21.85, Hym: 123.19±11.70; p=0.032) and the cerebellum (Con: 195.37±10.12, Hym: 154.74±9.81; p=0.018) (Table 1), as well as a decrease in the concentration of hippocampal MHPG - the main metabolite of noradrenaline (Con: 18.91±2.41, Hym: 10.84±2.26; p=0.024). The infected animals also demonstrated an increase in MHPG/NA turnover (Con: 0.23±0.11, Hym: 0.57±0.16; p=0.013) and a reduced level of serotonin in the striatum

compared to the control group (Con: 469.76±69.32, Hym: 231.06±14.08; p=0.001). The infected group also demonstrated a reduction of HVA level in the medulla oblongata (Con: 42.49±3.17, Hym: 25.33±3.85; p=0.018) and decrease in HVA/DA turnover (Con: 0.108±0.008, Hym: 0.063±0.009; p=0.003).

The infected rats also displayed an increase in glutamic acid concentration in the striatum (Con: 893.16±66.22, Hym: 1095.52±23.76; p=0.043) was observed (Table 2).

2.5. PCR results – a decrease in mRNA expression of hippocampal IL-6

The mRNA expression of the important proinflammatory factors in the brain structures was also determined using Real Time PCR. No significant differences were observed between groups for most tested factors; however, the rats infected with *H. diminuta* demonstrated decreased mRNA expression of hippocampal IL-6 (Con: 1.08±0.14, Hym: 0.63±0.05; $F_{(1,13)}$=8.426, p=0.043) (Table 3).

3. Discussion

The present study uses a rat model to analyze the impact of infection with *Hymenolepis diminuta* on behavioral parameters and neurotransmission in the host brain. The findings confirm the presence of a parasite-host interrelationship resulting in the modification of noradrenergic and serotonergic neurotransmission, manifested by changes in behavior and impaired locomotor activity in the infected animals. More specifically, the rats infected with *H. diminuta* exhibited inferior exploratory behavior and motor skills accompanied by increased thigmotaxis (the tendency to remain close to vertical surfaces) in the Open Field test. This slower movement speed may be caused by a decrease in noradrenalin level in the prefrontal cortex, striatum, and cerebellum. The decreased frequency in the central zone of the apparatus may indicate a higher level of anxiety in the infected rats. From an evolutionary perspective, anxiety has adaptive significance, allowing a creature to respond to

environmental changes, and brings some advantages such as improved danger recognition; when they are functioning properly, these mental states can modulate behavior and increase sensitivity to signs of potential danger. Anxiety is also a component of de-escalating strategies aimed at maintaining social homeostasis [43]. Infection was also found to decrease other important elements of animal activity, such as grooming; self-grooming is a maintenance behavior that provides the animal physiological status, comfort and appearance.

Contrary to previously published observations [1-17], our findings indicate that parasite infestation improved spatial memory, assessed in the Water Maze test, and recognition of new objects, in the NOR test, which may be related to changes in the levels of neurotransmitters. Williamson et al. [13, 18] showed that neonatal colonization of male rats by helminths prevented inflammation-induced cognitive impairments. Pups inoculated with *H. diminuta* and then injected with live *E. coli* and LPS did not show any memory disturbances in a fear conditioning task. In addition, maternal helminth inoculation prevented the increase of hippocampal IL-1 mRNA in neonatally-infected pups.

Differences in parasite biology may explain some discrepancies between our present results and those from previous studies on intestinal nematodes, as these can have a significant influence on microbiome structure and the immunomodulatory properties of parasite derived molecules. For example, infection with the intestinal trichostrongylid nematode *Heligmosomoides polygyrus* causes spatial learning deficit in male laboratory mice. Recent work has shown that mice with subclinical parasitic infections caused by the protozoan parasite *Eimeria vermiformis* and the gastrointestinal nematode *Heligmosomoides polygyrus* present impaired spatial learning and memory in Open Field and Water Maze testing, although the mechanism underlying this effect was not clear [19]. No visual, motor, or motivational impairments were apparent in the animals during the water maze test. In turn,

Braithwaite et al. [20] showed that infection with the nematode *Strongyloides ratti* did not affect learning or memory.

Intestinal helminths represent a biome capable of modulating the immune system in very different ways. While *H. dimunuta* is considered as a low pathogenic or non-pathogenic parasite, strong host immune responses are activated to prevent parasite establishment during invasion with haematophagic nematodes [21]. As a result, host immunity focuses on eliminating the parasite or confining it to minimize host damage. In addition, our results may differ from previous findings as the ability of rodents to learn a spatial or a discrimination task may also dependent on the type of parasite, the stage and level of infection, species of animal model and experimental protocol.

In the present study, the *H. diminuta* infected rats demonstrated lower striatal levels of serotonin than the control rats, which may be responsible for changes in animal behavior. Serotonin is a key neurotransmitter in the CNS and an important regulatory hormone controlling a broad range of physiological functions. 5-HT affects mood, sleep and anxiety, and peripherally, is involved in the modulation of gastrointestinal motility [22-24]. The enteric serotonergic system plays a principal role in the host-parasite interaction, impacting immunity and thereby the outcome of infection. It is believed that 5-HT is important for intestinal function, and approximately 90% of serotonin is formed in enterochromaffin cells in the gut in humans. Martin et al. [25] report that the central and peripheral pools of serotonin are anatomically separated because 5-HT cannot readily cross the blood-brain barrier.

When infected with helminth parasites, the mammalian gut triggers immune responses to eradicate the parasite, reduce tissue damage, and restore gut homeostasis. These are accompanied by discrete changes in the enteroendocrine and nervous systems, especially those concerning serotonergic signaling. Abdel Ghafar et al. [26] report a significant decrease

in serotonin, noradrenaline and dopamine content in the brain in mice experimentally infected with *Schistosoma mansoni*, *Toxocara canis* or *Trichinella spiralis*.

Parasite-induced changes in brain monoamine levels, especially brain serotonin signaling, may be associated with changes in host behavior. This adaptive mechanism probably greater parasite transmission to the predatory final hosts. As indicated in the literature, these changes may develop in two opposite directions, as parasite invasions have been associated with both increased and decreased serotonergic activity. Even so, invasions are known to influence the serotonergic signaling in the brain of the host, thus altering its behavior; indeed, Helland-Riise and co-workers [27] found that infection with brain-encysting trematodes of the species *Euhaplorchis californiensis* caused a decrease in raphe serotonergic neurotransmission in the intermediate host: the California killifish.

Euhaplorchis californiensis infection has also been observed to influence serotonin level by Mettrick and Cho [28], who report that higher 5-HT levels in the parasitized gut and blood than in uninfected animals. Moreover, serotonin is important in controlling worm behavior and metabolism [28-30]; it influences the migration of the worm by regulating of neuromuscular activity and the carbohydrate metabolism of its helminth.

Our data show that infestation by *H. diminuta* not only affects the serotonergic system but also influences noradrenergic neurotransmission. The noradrenergic system regulates various behavioral functions, including selective attention, memory storage and retrieval, vigilance and mood [31]. Noradrenergic neurons in the CNS originate mainly from the locus coeruleus and project to different brain areas, including the cortex, hippocampus, amygdala, thalamus, and hypothalamus. Deregulation of noradrenergic transmission in the brain may result in dementia or Alzheimer's Disease. One emerging idea proposes that Alzheimer's Disease is preceded by abnormal hyperactivation of the LC, resulting in over-secretion of NA in the cortex [32].

In the present experiment, *H. diminuta* invasion was associated with lower levels of NA in the prefrontal cortex and the cerebellum than in infected animals, thus influencing sympathetic nerve function and NA secretion. Similarly, other studies have found that intestinal inflammation in response to parasitic infection altered noradrenaline release. In the myenteric plexus of *Trichinella spiralis*-infected rats, a significant suppression of NA release was noted on the sixth day post infection, which persisted until 100 days post infection [33]. Sures et al [34] indicated that infections with the acanthocephalan *Moniliformis moniliformis* significantly reduced cortisol release and changed catecholamine concentrations in rats exposed to cadmium.

The impact of *H. diminuta* on the noradrenergic system may indicate its potential in studies of the role of parasitic infections on neurodegenerative diseases and suggest that it may offer some protective potential in this regard. Experimental studies performed by Williamson et al. [13, 18] suggest that infection may protect rats against memory loss, while Pillet et al. [35] note that abnormal noradrenergic neuron structure and function are closely related to the etiopathology of Alzheimer's Disease.

The significant decrease in noradrenaline and serotonin observed in our present experiment may explain the motor, behavioral and emotional changes observed in the rats infected with *H. diminuta*. Infection with this low pathogenic tapeworm appears to positively influence spatial memory and new object recognition; in addition, the infected animals develop a greater level of anxiety and move more carefully and slowly.

Regarding the expression of genes encoding proinflammatory cytokines, no major changes in the nervous system were connected with parasitic infection, apart from a reduction in the expression of IL-6, traditionally described as a proinflammatory cytokine. This may not only occur through the parasite exerting a direct effect on the host organism, but probably also due to changes it may make in the gut microbiome, which interacts with the immune system to

affect the CNS. Many researchers have emphasized the role of IL-6 in host defense against parasitic infections [36-38]. IL-6 participates in the innate and adaptive immune responses that protect the host from a variety of pathogens. For example, IL-6 is needed to develop protective immunity to *Toxoplasma gondii* during early infection, and plays a protective role against peripheral blood neutrophilia [39]. IL-6 is known to play a critical role in resistance to *T. gondii* and allows the host to control parasite replication. Silver et al. [40] propose that IL-6 is part of a *regulatory loop* that not only initiates inflammation but also limits immunological response in chronic conditions; as such, the lower expression of IL-6 observed in our study may hence be a result of adaptation to chronic infection. The aforementioned research suggests that the parasite exerts an influence on the host, which may lead to changes in the CNS functioning and immunological response.

No significant changes in the mRNA levels of other studied cytokines were observed in the CNS of rats infected with *H. diminuta* compared to controls. Transcripts for IL-1β, IL-10 and TNF-α were present in similar amounts in the CNS structures of the infected and parasite-free rats. However, to better determine the host immune response and the true effect of parasitic infection on cytokine levels, future studies should examine their concentration in the blood and CNS tissues by appropriate analytical methods.

Our results showed that infection with the tapeworm *H. diminuta* influences spatial memory and new object recognition. At the same time, the infected animals develop a greater level of anxiety and move more slowly. Visible changes in the exploratory and motor behavior and cognitive abilities of infected animals may be related to the decreased noradrenergic and serotonergic neurotransmission in the brain structures; however, although the precise nature of this process remains unclear, it may be associated with the modification of the intestinal microbiome. We believe that our results provide new data on the complex host-parasite crosstalk; this may serve as the basis for further research on the influence of parasites on brain

function and the development of diseases in which a key role may be played by noradrenergic system dysfunctions. Our research clearly indicates that the intestinal macrobiome can influence the behavior of the host. Therefore, in the near future it may turn out that the role of the macrobiome in the functioning of the nervous system may be just as significant as that of the microbiome.

4. Materials and Methods

4.1. Animals and treatment

Male Lewis rats, aged approximately three months, were used as definitive hosts for adult *H. diminuta*. All rats were kept in plastic cages in the Medical University of Warsaw laboratory animal facilities and were provided feed and water *ad libitum*.

Briefly, six-week-old *H. diminuta* cysticercoids were extracted from dissected *Tenebrio molitor* beetles under a microscope (magnification 100 x) and used to infect three-month-old rats by oral uptake of six cysticercoids of *H. diminuta* per rat. To verify the presence of adult parasites, fecal samples were taken from the animals and examined for the presence of tapeworm eggs under a microscope (magnification 400 x).

The rats were randomized into two equal groups: one group infected with *H. diminuta* (Hym, n=7) and an uninfected control group (Con, n=8). Both groups of rats were housed individually in plastic breeding cages in an air-conditioned room at 24 °C with 12 h dark-light cycle. Both infected animals and those from the control group received standard food (Labofeed, Kcynia, Poland) and tap water *ad libitum*. Parasite infestation was periodically monitored by fecal pellet examination using the fecal flotation test.

At 18 months old, the adult Lewis male rats were used for behavioral testing, after which the rats were sacrificed by decapitation. The body weights were recorded before sacrifice. Brain

tissues were immediately dissected, weighed, quickly frozen and stored at -80°C for future analysis.

All procedures were carried out in compliance with the directives of the National Research Council (NRC) Committee for the Update of the Guide for the Care and Use of Laboratory Animals (8th edition; National Academies Press, 2011) and approved by the Ethical Committee for Animal Experiments at Medical University of Warsaw. The ARRIVE guidelines 2.0 (Animal Research: Reporting of In Vivo Experiments) were implemented to ensure the highest quality of research standards.

4.2. Behavioral tests

A detailed experimental diagram is shown in Figure 3.

4.2.1. Open Field (OF) test

The Open Field (OF) test allows quantification of animal exploration, as well as various locomotor parameters, such as total distance traveled or time spent on moving. OF also allowed the animals to become habituated to the apparatus, which was subsequently used during the Novel Object Recognition (NOR) test performed the following day. This attitude shortened the time needed for the activities and reduced the discomfort of the animals caused by the additional procedures.

For the OF test, a rat was placed in the center of a gray open field bounded by walls measuring 100 cm x 100 cm x 40 cm (length x width x high). The test apparatus was located in an acoustically-isolated experimental room illuminated by diffused light. On the test day, each rat was placed at the center of arena; the locomotor activity of the exploring animal was monitored for eight minutes with a video camera located above the OF arena and recorded with the EthoVision XT10 software (Noldus). The behavior of the animal was scored by

observers blinded to the experimental plan. In order to eliminate odor traces, the field had been cleaned with 70% alcohol before each animal was tested.

4.2.2. Novel Object Recognition (NOR) test

The Novel Object Recognition (NOR) test is used to assess the learning and memory processes in animals and helps in the assessment of neuropsychological changes. The study was carried out in the apparatus used for the OF test; however, the it was modified with the addition of two objects for exploration. After each trial, the objects used in the experiment and the testing box itself were disinfected with a 70% solution of ethanol. The test consisted of three stages: habituation, in this case fulfilled by the OF test, familiarization and choice phases. The study was conducted individually for each animal. During the familiarization phase, the animals were placed against the wall opposite another wall where two identical objects (towers made of Lego blocks) marked as A1 and A2 were placed (located at opposite corners of the apparatus). The animals were allowed to freely explore the apparatus for three minutes. On the next day, one of the objects was replaced with another unknown object (object B - glass bottle). New and familiar items were presented in different colors, structures and shapes. Object exploration was classified as the rat placing its head at a distance of 2 cm from any object. During the experiment, the time spent exploring individual objects during each phases and the total time spent exploring both objects were measured, and the Discrimination Index was calculated for the choice phase as the difference between the time (t) taken to explore the novel and known objects [DI=(tB-tA1)/(tB+tA1)]. The Index of Global Habituation [GHI=(tA1+tA2)/(tB+tA1)], which compares the time spent studying two subjects during the familiarization phase with the time spent during the choice phase, was also estimated. The Recognition Index, the time spent on researching the new object in relation to the total exploration of the objects [RI = tB/(tB+tA1)], was also determined.

4.2.3. Water Maze (WM) test

A modified version of the Water Maze (WM) test was used to evaluate the influence of *H. diminuta* infestation on learning and memory in rats. To reduce the stress and natural aversion of animals to the water, the rats were habituated to the experimental conditions 24 hours before the behavioral tests. The WM test was performed in a circular pool (150 cm in diameter; 50 cm high) filled with water (30 cm height) maintained at 23°C and divided conventionally into four quadrants (Southeast – SE, Southwest – SW, Northeast – NE, Northwest – NW). The maze was located in a room and was surrounded by several objects as spatial coordinates e.g., shelves, screens and posters.

During the water acquisition phase, the rats were trained for four consecutive days (four trials per day) to locate a hidden transparent round, plexiglass platform (10 cm diameter) submerged 1 cm below the water surface in the middle of the SE sector. In each trial, the rat was randomly placed into the pool facing the wall at an equidistant cardinal points in a pseudorandom schedule. If the rat found the platform, it could stay there for 15 seconds to sufficiently assess distal cues. When the animal failed to find the platform within 60 seconds, the experimenter manually guided the rat to the platform for 15 s. Then each rat was removed from the tank, dried by wiping with a cloth, and returned to its holding cage.

During the spatial memory test assessed on the fifth day (trial 17, Memory Test I) the platform was removed from the pool, and the animals searched for the absent platform for 60 seconds.

Two days later (day 8, trials 18-21), a Remainder Test was conducted. The platform was placed again in the SE sector, and the rats recalled the location of the underwater goal.

A reversal trial was carried out on day 9 (trials 22-25). The platform was transferred to the center of the NW sector and the animals learned to find its new position. On day 10 (trial 26,

Memory Test II) the platform was removed again, and the rats swam only once for 60 seconds, searching for the absent platform.

Sensorimotor skills and motivation were assessed on day 11 during a cued task (visible platform task). In this task, the daily training session consisted of four attempts. The visible platform was located 1 cm above the water level and was moved successively between four sectors. Three randomly-assigned starting points were located at the perimeter of the pool, in the center points of the sectors without the platform. The test was ended when the rat reached the platform, or after 60 seconds.

Latencies to find the platform, a number of visits in a target area and the time spent in the goal quadrant, swim paths and velocity were recorded using a video camera and EthoVision XT10 software (Noldus).

4.3. HPLC

HPLC was used to estimate the concentrations of monoamines and amino acids in selected rat CNS tissues: hippocampus, prefrontal cortex, striatum, hypothalamus, cerebellum and medulla oblongata.

4.3.1. Sample preparation

Before analysis, brain samples were homogenized using ultrasonic cell disrupter (VirSonic 60; VirTis, USA) in a mixture (1000 µl) containing ice-cold 0.1 N perchloric acid (HClO4) and 0.05 mM ascorbic acid, and then centrifuged to precipitate proteins (Heraeus Labofuge 400 R, Heraeus Instruments, Germany; centrifugation conditions: speed of 13,000 x g, time of 15 min, temperature of 4ºC). After filtration using syringe membrane filters with pore size 0.2 µm (Puradisc; Whatman, UK), the supernatant was collected and used to perform biochemical analyses.

4.3.2. Assay of monoamine concentrations

To determine the concentration of serotonin (5-HT) and its metabolite 5-hydroxyindoleacetic acid (5-HIAA), dopamine (DA) and its metabolites 3,4-dihydroxyphenylacetic acid (DOPAC) and homovanillic acid (HVA), and noradrenaline (NA) and its metabolite 3-methoxy-4-hydroxyphenylglycol (MHPG) a 20 µl aliquot of supernatant was injected into the HPLC apparatus with electrochemical detection (HPLC-ED) system.

The HPLC system consisted of a delivery pump (Mini-Star K-500; Knauer, Berlin Germany), an autosampler automatic injector (LaChrom L-7250; Merck-Hitachi, Darmstadt/Tokyo, Germany/Japan), an electrochemical detector (L-3500A; Merck-Recipe, Darmstadt/Munich, Germany) set at a potential of +0.8 V *vs* an Ag/AgCl reference electrode. The mobile phase comprised 32 mM sodium phosphate buffer (Sigma-Aldrich, St. Louis, MO, USA), 34 mM citric acid buffer (Sigma-Aldrich, USA), 1 mM octane sulfonic acid buffer (Sigma-Aldrich, USA), 54 µM ethylenediaminetetraacetic acid (EDTA) buffer (Sigma-Aldrich, USA) in ultrapure water (18 MΩ·cm) containing 12% methanol solution (Merck, Germany). Monoamines were separated using EC 250/4 Nucleosil 100-5 C18AB (250 mm length x 4 mm internal diameter, 5 µm particle size, 100Å) HPLC analytical column (Macherey-Nagel, Germany) and mobile phase flow rate maintained at 0.8 ml/min. Chromatograms were recorded and integrated by use of the computerized data acquisition Clarity software (version 5.0; DataApex, Prague, Czech Republic). Samples were quantified by comparison with standard solutions (external calibration). All used monoamines standards were purchased from Sigma-Aldrich, USA. The final amount of monoamines/metabolites in the tissue sample was expressed as pg/mg wet tissue.

4.3.3. Assay of Amino acid concentrations

High Performance Liquid Chromatography with electrochemical detection (HPLC-ED) was used to determine the concentration of the following amino acids: glutamic acid (GLU), aspartic acid (ASP) gamma-aminobutyric acid (GABA), alanine (ALA), histidine (HIS), serine (SER), arginine (ARG), threonine (THR), glutamine (GLN) and taurine (TAU) in the medium. The chromatograph system consisted of an electrochemical detector with glassy-carbon working electrode (EC 3000, Merck), the autosampler (Primade 1210, Hitachi) and the pump (Primade 1110, Hitachi). The mobile phase comprised 45 mM disodium phosphate (Sigma–Aldrich) and 0.15 mM ethylenediaminetetraacetic acid (EDTA; Sigma–Aldrich) with 24% methanol (Merck). To obtain agents for derivatisation o-phthaldialdehyde (OPA, 22 mg, Fluka) was diluted in 0.5 ml of 1 M sodium sulphite, 0.5 l of methanol, 0.9 ml of 0.1 M sodium tetraborate buffer (adjusted to pH 10.4 using 5 M sodium hydroxide). The preparation of the mobile phase and the derivatising agents were based on the method developed by Rowley et al. [41] with minor modifications. Prior to application onto the column, the derivatising agent (20 μl) was reacted with amino acid standard and with supernatant samples for 15 min at room temperature. Separation of amino acids was achieved with a reverse phase column (Luna; 5 μm C18(2) 100A; 250 mm length × 4 mm internal diameter; Phenomenex) and mobile phase flow rate maintained at 0.8 ml/min. Samples were quantified by comparison with standard solutions (external calibration) and concentrations calculated with Primade (Merck). All standards were purchased from Sigma–Aldrich. The final amount of amino acids in the tissue sample was expressed as ng/mg wet tissue.

4.4. Real-time polymerase chain reaction (Real-Time PCR)

The mRNA expression of pro and anti-inflammatory cytokines TNF-α, IL-1β, IL-6 and IL-10 were assessed using real-time PCR. Total RNA was extracted from the tissue using the TRI Reagent (Sigma-Aldrich, USA), in accordance with the manufacturer's instructions. The

concentration of isolated RNA was determined spectrophotometrically at 260 nm. Single-strand cDNA was synthesized from total RNA using PrimeScript™ RT reagent Kit (Takara Bio Inc, Japan) in a Labcycler (SensoQuest GmbH, Germany). The incubation conditions for reverse transcription were 15 min at 37°C followed by 5 s at 85°C. Following the reverse transcription reaction, cDNA products were stored at –20 °C until use.

Real-time PCR was conducted using a Rotor Gene Q 5plex HRM System (QIAGEN Research, Inc., Netherlands). The cDNA was amplified with gene-specific primers designed using Primer BLAST software (NCBI, USA) (Table 4). The housekeeping gene (glyceraldehyde 3-phosphate dehydrogenase, GAPDH) was used to normalize gene expression levels.

The PCR mixtures contained 1 μl of previously reverse-transcribed cDNA along with 10 μl of FastStart Essential DNA Green Master (Roche Molecular Systems, Inc., USA) and 1.25 μl (10 μM) of each primer in a total reaction volume of 20 μl. The amplification protocol was as follows: initial denaturation at 95°C for 10 min; 40 cycles at 95°C for 15 s, 58°C for 15 s, and 72°C for 15 s. The reaction melting-curve analysis was applied to all reactions to ensure the consistency and specificity of the amplified product. All amplifications were carried out in duplicate. The relative expression of the genes was determined according to Pfaffl [42].

4.5. Statistical analysis

The results were analyzed using STATISTICA 10.0 software (StatSoft, Poland). Behavioral training was analyzed with repeated measurements ANOVA to analyze latencies in the water maze between treatment groups over several training days. Memory probe tests and other behavioral parameters were analyzed with the Newman-Keuls test. Neurotransmitter concentration and mRNA expression for the target genes were analyzed with the Mann-

Whitney U-test, comparing control and infected groups. All the data are presented as mean ± SEM. The results were considered statistically significant at $p<0.05$.

5. Acknowledgments

All co-authors approved the final version of the manuscript. All applicable international, national, and/or institutional guidelines for the care and use of animals were followed.
The authors declare that they have no competing interests. This research was partially supported by the National-Science-Center-Poland (Grant Number 2014/13/B/NZ6/00881).
Project implemented with CePT infrastructure financed by the European Union - the European Regional Development Fund within the Operational Programme "Innovative economy" for 2007-2013".

References

1. Bień J, Sałamatin R, Sulima A, Savijoki K, Bruce Conn D, Näreaho A, Młocicki D. Mass spectrometry analysis of the excretory-secretory (E-S) products of the model cestode Hymenolepis diminut a reveals their immunogenic properties and the presence of new E-S proteins in cestodes. Acta Parasitol. 2016 Mar;61(2):429-42. doi: 10.1515/ap-2016-0058. PMID: 27078671.
2. Młocicki D, Sulima A, Bień J, Näreaho A, Zawistowska-Deniziak A, Basałaj K, Sałamatin R, Conn DB, Savijoki K. Immunoproteomics and Surfaceomics of the Adult Tapeworm Hymenolepis diminuta. Front Immunol. 2018 Nov 12;9:2487. doi: 10.3389/fimmu.2018.02487. PMID: 30483248; PMCID: PMC6240649.
3. McKenney EA, Williamson L, Yoder AD, Rawls JF, Bilbo SD, Parker W. Alteration of the rat cecal microbiome during colonization with the helminth Hymenolepis diminuta. Gut

Microbes. 2015;6(3):182-93. doi: 10.1080/19490976.2015.1047128. PMID: 25942385; PMCID: PMC4615828.

4. Zawistowska-Deniziak A, Basałaj K, Strojny B, Młocicki D. New Data on Human Macrophages Polarization by Hymenolepis diminuta Tapeworm-An In Vitro Study. Front Immunol. 2017 Feb 20;8:148. doi: 10.3389/fimmu.2017.00148. PMID: 28265273; PMCID: PMC5316519.

5. Jirků Pomajbíková K, Jirků M, Levá J, Sobotková K, Morien E, Parfrey LW. The benign helminth Hymenolepis diminuta ameliorates chemically induced colitis in a rat model system. Parasitology. 2018 Sep;145(10):1324-1335. doi: 10.1017/S0031182018000896. Epub 2018 Jun 18. PMID: 29909781.

6. Rhee SH, Pothoulakis C, Mayer EA. Principles and clinical implications of the brain-gut-enteric microbiota axis. Nat Rev Gastroenterol Hepatol. 2009 May;6(5):306-14. doi: 10.1038/nrgastro.2009.35. PMID: 19404271; PMCID: PMC3817714.

7. Carabotti M, Scirocco A, Maselli MA, Severi C. The gut-brain axis: interactions between enteric microbiota, central and enteric nervous systems. Ann Gastroenterol. 2015 Apr-Jun;28(2):203-209. PMID: 25830558; PMCID: PMC4367209.

8. Kuwahara A, Matsuda K, Kuwahara Y, Asano S, Inui T, Marunaka Y. Microbiota-gut-brain axis: enteroendocrine cells and the enteric nervous system form an interface between the microbiota and the central nervous system. Biomed Res. 2020;41(5):199-216. doi: 10.2220/biomedres.41.199.

9. Karakan T, Ozkul C, Küpeli Akkol E, Bilici S, Sobarzo-Sánchez E, Capasso R. Gut-Brain-Microbiota Axis: Antibiotics and Functional Gastrointestinal Disorders. Nutrients. 2021 Jan 27;13(2):389. doi: 10.3390/nu13020389. PMID: 33513791; PMCID: PMC7910879.

10. Heil M. 2016. Host Manipulation by Parasites: Cases, Patterns, and Remaining Doubts. Front. Ecol. Evol. 4:80. doi: 10.3389/fevo.2016.00080.

11. Melon A, Wang A, Phan V, McKay DM. Infection with Hymenolepis diminuta is more effective than daily corticosteroids in blocking chemically induced colitis in mice. J Biomed Biotechnol. 2010;2010:384523. doi: 10.1155/2010/384523. PMID: 20011066; PMCID: PMC2789531.

12. Abdoli A, Mirzaian Ardakani H. Potential application of helminth therapy for resolution of neuroinflammation in neuropsychiatric disorders. Metab Brain Dis. 2020 Jan;35(1):95-110. doi: 10.1007/s11011-019-00466-5. Epub 2019 Jul 27. PMID: 3135253.

13. Williamson LL, McKenney EA, Holzknecht ZE, Belliveau C, Rawls JF, Poulton S, Parker W, Bilbo SD. Got worms? Perinatal exposure to helminths prevents persistent immune sensitization and cognitive dysfunction induced by early-life infection. Brain Behav Immun. 2016 Jan;51:14-28. doi: 10.1016/j.bbi.2015.07.006. Epub 2015 Jul 7. PMID: 26162711.

14. Wegener Parfrey L, Jirků M, Šíma R, Jalovecká M, Sak B, Grigore K, Jirků Pomajbíková K. A benign helminth alters the host immune system and the gut microbiota in a rat model system. PLoS One. 2017 Aug 3;12(8):e0182205. doi: 10.1371/journal.pone.0182205. PMID: 28771620; PMCID: PMC5542714.

15. Kannan G, Moldovan K, Xiao JC, Yolken RH, Jones-Brando L, Pletnikov MV. Toxoplasma gondii strain-dependent effects on mouse behaviour. Folia Parasitol (Praha). 2010 Jun;57(2):151-5. doi: 10.14411/fp.2010.019. PMID: 20608478.

16. Reverchon F, Mortaud S, Sivoyon M, Maillet I, Laugeray A, Palomo J, Montécot C, Herzine A, Meme S, Meme W, Erard F, Ryffel B, Menuet A, Quesniaux VFJ. IL-33 receptor ST2 regulates the cognitive impairments associated with experimental cerebral malaria. PLoS Pathog. 2017 Apr 27;13(4):e1006322. doi: 10.1371/journal.ppat.1006322. PMID: 28448579; PMCID: PMC5407765.

17. Pan SC, Ward DV, Yin Y, Hu Y, Elfawal MA, Clark RE, Aroian RV. Cognitive and Microbiome Impacts of Experimental Ancylostoma ceylanicum Hookworm Infections in Hamsters. Sci Rep. 2019 May 27;9(1):7868. doi: 10.1038/s41598-019-44301-4. PMID: 31133690; PMCID: PMC6536493.

18. Williamson L, McKenney E, Parker W, Bilbo S. 30. Biome reconstitution as a novel mechanism of preventing neonatal infection-induced cognitive dysfunction. Brain, Behavior, and Immunity Volume 40, Supplement, 2014, Page e9,ISSN 0889-1591, https://doi.org/10.1016/j.bbi.2014.06.050.

19. Kavaliers M, Colwell DD. Reduced spatial learning in mice infected with the nematode, Heligmosomoides polygyrus. Parasitology. 1995 Jun;110 (Pt 5):591-7. doi: 10.1017/s0031182000065318. PMID: 7596642.

20. Braithwaite VA, Salkeld DJ, McAdam HM, Hockings CG, Ludlow AM, Read AF. Spatial and discrimination learning in rodents infected with the nematode Strongyloides ratti. Parasitology. 1998 Aug;117 (Pt 2):145-54. doi: 10.1017/s003118209800290x. PMID: 9778637.

21. Grencis RK, Humphreys NE, Bancroft AJ. Immunity to gastrointestinal nematodes: mechanisms and myths. Immunol Rev. 2014 Jul; 260(1):183-205. doi: 10.1111/imr.12188. PMID: 24942690; PMCID: PMC4141702.

22. Naoi M, Maruyama W, Shamoto-Nagai M. Type A monoamine oxidase and serotonin are coordinately involved in depressive disorders: from neurotransmitter imbalance to impaired neurogenesis. J Neural Transm (Vienna). 2018 Jan;125(1):53-66. doi: 10.1007/s00702-017-1709-8. Epub 2017 Mar 14. PMID: 28293733.

23. Israelyan N, Del Colle A, Li Z, Park Y, Xing A, Jacobsen JPR, Luna RA, Jensen DD, Madra M, Saurman V, Rahim R, Latorre R, Law K, Carson W, Bunnett NW, Caron MG, Margolis KG. Effects of Serotonin and Slow-Release 5-Hydroxytryptophan on

Gastrointestinal Motility in a Mouse Model of Depression. Gastroenterology. 2019 Aug;157(2):507-521.e4. doi: 10.1053/j.gastro. 2019.04.022. Epub 2019 May 7. PMID: 31071306; PMCID: PMC6650329.

24. Jones LA, Sun EW, Martin AM, Keating DJ. The ever-changing roles of serotonin. Int J Biochem Cell Biol. 2020 Aug;125:105776. doi: 10.1016/j.biocel.2020.105776. Epub 2020 May 29. PMID: 32479926.

25. Martin AM, Young RL, Leong L, Rogers GB, Spencer NJ, Jessup CF, Keating DJ. The Diverse Metabolic Roles of Peripheral Serotonin. Endocrinology. 2017 May 1;158(5):1049-1063. doi: 10.1210/en.2016-1839. PMID: 28323941.

26. Abdel Ghafar AE, Elkowrany SE, Salem SA, Menaisy AA, Fadel WA, Awara WM. Effect of some parasitic infection on neurotransmitters in the brain of experimentally infected mice before and after treatment. J Egypt Soc Parasitol. 1996 Aug;26(2):497-508. PMID: 8754657.

27. Helland-Riise SH, Vindas MA, Johansen IB, Nadler LE, Weinersmith KL, Hechinger RF, Øverli Ø. Brain-encysting trematodes (Euhaplorchis californiensis) decrease raphe serotonergic activity in California killifish (Fundulus parvipinnis). Biol Open. 2020 Jul 8;9(7):bio049551. doi: 10.1242/bio.049551. PMID: 32439741; PMCID: PMC7358127.

28. Mettrick DF, Cho CH. Migration of Hymenolepis diminuta (Cestoda) and changes in 5-HT (serotonin) levels in the rat host following parenteral and oral 5-HT administration. Can J Physiol Pharmacol. 1981 Mar;59(3):281-6. doi: 10.1139/y81-044. PMID: 7225951.

29. Li Y, Zhao Y, Huang X, Lin X, Guo Y, Wang D, Li C, Wang D. Serotonin control of thermotaxis memory behavior in nematode Caenorhabditis elegans. PLoS One. 2013 Nov 1;8(11):e77779. doi: 10.1371/journal.pone.0077779. PMID: 24223727; PMCID: PMC3815336.

30. Mori H, Ashida K, Shidara H, Nikai T, Hotta K, Oka K. Serotonin modulates behavior-related neural activity of RID interneuron in Caenorhabditis elegans. PLoS One. 2019 Dec 4;14(12):e0226044. doi: 10.1371/journal.pone.0226044. PMID: 31800640; PMCID: PMC6892467.

31. Sara SJ. The locus coeruleus and noradrenergic modulation of cognition. Nat Rev Neurosci. 2009 Mar;10(3):211-23. doi: 10.1038/nrn2573. Epub 2009 Feb 4. PMID: 19190638.

32. Ross JA, Gliebus G, Van Bockstaele EJ. Stress induced neural reorganization: A conceptual framework linking depression and Alzheimer's disease. Prog Neuropsychopharmacol Biol Psychiatry. 2018 Jul 13;85:136-151. doi: 10.1016/j.pnpbp.2017.08.004. Epub 2017 Aug 10. PMID: 28803923; PMCID: PMC5809232.

33. Swain MG, Blennerhassett PA, Collins SM. Impaired sympathetic nerve function in the inflamed rat intestine. Gastroenterology. 1991 Mar;100(3):675-82. doi: 10.1016/0016-5085(91)80011-w. PMID: 1847118.

34. Sures B, Scheef G, Klar B, Kloas W, Taraschewski H. Interaction between cadmium exposure and infection with the intestinal parasite Moniliformis moniliformis (Acanthocephala) on the stress hormone levels in rats. Environ Pollut. 2002;119(3):333-40. doi: 10.1016/s0269-7491(01)00340-2. PMID: 12166667.

35. Pillet LE, Taccola C, Cotoni J, Thiriez H, André K, Verpillot R. Correlation between cognition and plasma noradrenaline level in Alzheimer's disease: a potential new blood marker of disease evolution. Transl Psychiatry. 2020 Jul 3;10(1):213. doi: 10.1038/s41398-020-0841-7.

36. Mezioug D, Touil-Boukoffa C. Interleukin-17A correlates with interleukin-6 production in human cystic echinococcosis: a possible involvement of IL-17A in immunoprotection

against Echinococcus granulosus infection. Eur Cytokine Netw. 2012 Jul-Sep;23(3):112-9. doi: 10.1684/ecn.2012.0314. PMID: 23009764.

37. Ponce NE, Carrera-Silva EA, Pellegrini AV, Cazorla SI, Malchiodi EL, Lima AP, Gea S, Aoki MP. Trypanosoma cruzi, the causative agent of Chagas disease, modulates interleukin-6-induced STAT3 phosphorylation via gp130 cleavage in different host cells. Biochim Biophys Acta. 2013 Mar;1832(3):485-94. doi: 10.1016/j.bbadis.2012.12.003. Epub 2012 Dec 16. PMID: 23253440.

38. Rose-John S, Winthrop K, Calabrese L. The role of IL-6 in host defence against infections: immunobiology and clinical implications. Nat Rev Rheumatol. 2017 Jul;13(7):399-409. doi: 10.1038/nrrheum.2017.83. Epub 2017 Jun 15. PMID: 28615731.

39. Jebbari H, Roberts CW, Ferguson DJ, Bluethmann H, Alexander J. A protective role for IL-6 during early infection with Toxoplasma gondii. Parasite Immunol. 1998 May;20(5):231-9. doi: 10.1046/j.1365-3024.1998.00152.x. PMID: 9651924.

40. Silver JS, Stumhofer JS, Passos S, Ernst M, Hunter CA. IL-6 mediates the susceptibility of glycoprotein 130 hypermorphs to Toxoplasma gondii. J Immunol. 2011 Jul 1;187(1):350-60. doi: 10.4049/jimmunol.1004144. Epub 2011 May 23. PMID: 21606248; PMCID: PMC3119722.

41. Rowley HL, Martin KF, Marsden CA. Determination of in vivo amino acid neurotransmitters by high-performance liquid chromatography with o-phthalaldehyde-sulphite derivatisation. J Neurosci Methods. 1995 Mar;57(1):93-9. doi: 10.1016/0165-0270(94)00132-z. PMID: 7791370.

42. Pfaffl MW. A new mathematical model for relative quantification in real-time RT-PCR. Nucleic acids research. 2001;29(9):e45.

43. Bateson M, Brilot B, Nettle D. Anxiety: an evolutionary approach. Can J Psychiatry. 2011 Dec;56(12):707-15. doi: 10.1177/070674371105601202. PMID: 22152639.

Figure legends

Fig. 1. Main Open Field parameters for the control rats (Con, n=8) and rats infected with H. diminuta (Hym, n=7). A,B,C,D

* Hym vs Con, $p<0.05$ (Newman-Keuls test)

*** Hym vs Con, $p<0.005$ (Newman-Keuls test)

Fig. 2. Results from the Morris water maze test. Effect of parasite infection on crossings under previous platform position in SE on first memory test (A) and under previous (B) and new platform position in NW (C) during second memory probe trial. Each column represents the mean ± standard error of mean (SEM).

** Hym vs Con, $p<0.01$ (Newman-Keuls test)

Fig. 3. The graphical course of the experiment (created with BioRender.com).

Table 1. Monoamine and metabolite levels in the CNS structures of control rats (Con, n=8) and rats infected with *H. diminuta* (Hym, n=7).

*** Hym *vs* Control, p<0.05 (Mann-Whitney-U test)**

**** Hym *vs* Con, p<0.01 (Mann-Whitney-U test)**

Monoamine and metabolite levels in the CNS structures pg/g tissue (mean ± SEM)												
	**hippocampus**		**prefrontal cortex**		**striatum**		**hypothalamus**		**cerebellum**		**medulla oblongata**	
	**Con**	**Hym**	**Con**	**Hym**	**Con**	**Hym**	**Con**	**Hym**	**Con**	**Hym**	**Con**	**Hym**
**NA**	215.64±33.06	164.04±25.53	**148.34±21.85**	**123.19±11.70***	255.99±14.45	49.78±11.87	1221.19±126.28	916.93±208.60	**195.37±10.12**	**154.74±9.81***	302.46±26.11	310.39 ±37.57
MHPG	**18.91 ±2.41**	**10.84±2.26***	29.34±4.72	21.87±4.22	17.13±3.57	22.34±3.95	1.98±0.87	1.41±1.09	1.32±0.40	0.53±0.22	1.04±0.28	0.88±0.44
MHPG/NA	0.11 ±0.03	0.09±0.03	0.18±0.03	0.19±0.05	**0.23±0.11**	**0.57±0.16***	n.d.	n.d.	n.d.	n.d.	n.d.	n.d.
**DA**	800.36 ±65.11	872.31±130.09	427.66±39.56	428.31±24.87	892.33±97.25	759.67±39.13	594.01±31.82	538.19±103.11	37.94±1.70	38.41±4.42	394.76±14.35	397.98±31.59
**DOPAC**	206.59 ±52.76	250.26±91.70	252.54±34.42	227.91±36.37	1927.23±394.88	2824.79±501.73	536.15±54.85	536.32±32.77	24.67±2.11	22.75±2.22	35.01±4.39	36.96±5.13
**DOPAC/DA**	0.30 ±0.09	0.42±0.20	0.63±0.12	0.53±0.08	2.36±0.57	3.77±0.73	0.92±0.10	7.50±6.58	0.66±0.07	0.63±0.10	0.09±0.01	0.10±0.01
HVA	15.01 ±7.85	50.90±25.98	n.d.	n.d.	454.45±91.53	664.13±116.06	48.16±6.55	65.63±15.08	35.02±2.14	30.40±2.98	**42.49±3.17**	**25.33±3.85***
**HVA/DA**	0.02 ±0.01	0.09±0.05	n.d.	n.d.	0.56±0.13	0.87±0.16	0.08±0.01	1.85±1.77	0.94±0.08	0.87±0.16	**0.11±0.01**	**0.06±0.01****
**5-HT**	298.09 ±53.41	319.06±48.72	114.25±37.81	163.95±31.59	**469.76±69.32**	**231.06±14.08****	797.04±63.52	619.86±94.52	44.23±2.65	47.83±2.96	487.25±26.08	527.65±36.09
**5-HIAA**	158.52 ±122.26	250.99±20.40	n.d.	n.d.	4128.69±977.71	3401.25±632.38	258.27±37.51	178.66±26.40	9.23±1.13	5.91±0.82	45.69±1.75	47.41±3.00
**5-HIAA/5-HT**	0.69 ±0.48	1.25±0.95	n.d.	n.d.	10.55±3.17	15.55±3.41	0.32±0.04	0.30±0.03	0.21±0.02	0.13±0.02	0.09±0.00	0.09±0.01
**3-MT**	n.d.	n.d.	n.d.	n.d.	306.56±70.02	283.62±51.04	24.13±8.38	18.89±7.04	2.60±0.68	7.38±3.28	22.67±9.57	26.76±6.64

Table 2. Aminoacid levels in the CNS structures of control rats (Con, n=8) and rats infected with *H. diminuta* (Hym, n=7).

*** Hym *vs* Control, p<0.05 (Mann-Whitney-U test)**

Aminoacids level in the CNS structures ng/mg tissue (mean ± SEM)												
	**hippocampus**		**prefrontal cortex**		**striatum**		**hypothalamus**		**cerebellum**		**medulla oblongata**	
	**Con**	**Hym**	**Con**	**Hym**	**Con**	**Hym**	**Con**	**Hym**	**Con**	**Hym**	**Con**	**Hym**
**Glutamic acid**	772.84±71.56	666.26±55.35	1151.39±63.82	1118.50±51.31	**893.16±66.22**	**1095.52±23.76***	938.87±68.80	898.55±62.10	847.61±105.00	903.51±156.78	602.52±18.54	643.38±31.14
**Taurine**	538.75±72.55	470.0 3±83.91	1100.39±91.52	999.51±52.92	1417.30±136.37	1450.37±69.79	480.00±31.00	544.74±135.29	552.25±67.19	519.70±97.25	262.47±5.63	261.39±6.93
**Alanine**	56.48±2.62	56.27±4.78	115.01±4.42	106.72±5.23	87.28±5.41	106.26±7.44	44.86±2.40	109.37±63.26	41.53±5.25	42.45±7.90	52.61±2.88	52.55±2.91
**γ-Aminobutyric acid**	662.77±67.52	603.37±33.10	782.79±24.28	736.27±22.93	1198.92±165.53	835.02±48.99	904.14±62.21	721.52±133.25	225.24±28.63	231.91±43.39	238.60±12.28	231.67±12.22
**Aspartic acid**	374.50±18.85	329.13±25.47	550.94±22.52	538.21±62.24	392. 93±27.80	373.30±34.41	315.72±19.30	294.96±33.67	167.29±23.38	145.52±29.94	277.22±16.41	249.32±12.84
**Histidine**	15.06±0.55	12.91±0.66	22.21±1.46	19.88±1.46	20.68±0.91	22.78±2.87	15.91±1.14	19.32±3.92	10.15±1.22	10.13±1.73	9.31±0.45	9.09±0.39
**Serine**	82.45±8.39	76.75±10.38	120.31±7.41	116.83±6.41	132.11±4.16	156.58±18.56	57.73±3.35	59.55±5.77	44.43±5.46	43.94±7.35	46.86±1.62	44.75±0.97
**Glutamine**	698.13±40.67	642.38±59.33	905.43±56.57	937.99±50.91	966.04±50.69	1007.98±47.65	905.70±54.57	821.25±67.41	699.70±90.71	739.53±135.04	553.82±12.19	577.81±25.19
**Arginine**	133.16±9.88	150.56±14.02	106.52±4.36	112.64±6.7	236.02±14.38	218.93±11.42	65.77±5.87	74.31±11.97	39.13±7.39	36.46±8.31	77.20±6.31	92.38±7.59
**Threonine**	73.29±7.00	87.79±3.68	58.78±3.92	53.16±3.39	65.10±2.44	63.60±3.98	53.19±3.05	52.55±7.23	45.39±4.93	42.41±7.03	45.18±2.11	40.32±2.57

Table 3. Real-time quantitative RT-PCR analyses for control rats (Con, n=8) and rats infected with *H. diminuta* (Hym, n=7). Gene expression levels represent the mRNA expression levels relative to the control. Values represent the means ± SEM

*** Hym *vs* Control, p<0.05 (Newman-Keuls test)**

	Relative expression (± SEM)							
	**Hipocampus**		**Prefrontal cortex**		**Striatum**		**Cerebellum**	
**Target gene**	**Con**	**Hym**	**Con**	**Hym**	**Con**	**Hym**	**Con**	**Hym**
IL-6	**1.08 ± 0.14**	**0.63 ± 0.05***	1.10 ± 0.18	1.37 ± 0.31	1.04 ± 0.10	1.50 ± 0.30	1.05 ± 0.35	1.20 ± 0.17
IL-10	1.28 ± 0.29	0.65 ± 0.34	1.11 ± 0.20	1.57 ± 0.19	1.09 ± 0.18	2.33 ± 1.25	1.11 ± 0.53	1.48 ± 0.35
IL-1β	1.18 ± 0.31	0.91 ± 0.12	1.04 ± 0.13	1.03 ± 0.11	1.05 ± 0.13	1.53 ± 0.40	1.07 ± 0.14	1.13 ± 0.19
TNF-α	1.08 ± 0.14	0.87 ± 0.22	1.15 ± 0.23	1.10 ± 0.15	1.01 ± 0.05	1.41 ± 0.22	1.05 ± 0.12	0.96 ± 0.18

Table 4. Sequences of gene specific primers used in Real Time PCR (F-forward, R-reverse)

Target gene	Sequences of gene-specyfic primers (5’>3’)		Predicted product lenght (bp)
GAPDH	F<br>R	GCTCTCTGCTCCTCCCTGTTCT<br>TCCGATACGGCCAAATCCGTT	111
IL-6	F<br>R	GCCAGTTGCCTTCTTGGGAC<br>CCTCTGTGAAGTCTCCTCTCCG	83
IL-10	F<br>R	CCATTCCATCCGGGGTGACA<br>TCAGCTCTCGGAGCATGTGG	71
IL-1β	F<br>R	ACTCGTGGGATGATGACGACC<br>GGTCAGACAGCACGAGGCAT	102
TNF-α	F<br>R	GTGGCTCTGGGTCCAACTCC<br>CCGCAATCCAGGCCACTACT	91

Figure 1

Click here to access/download;Figure;Fig1.tif

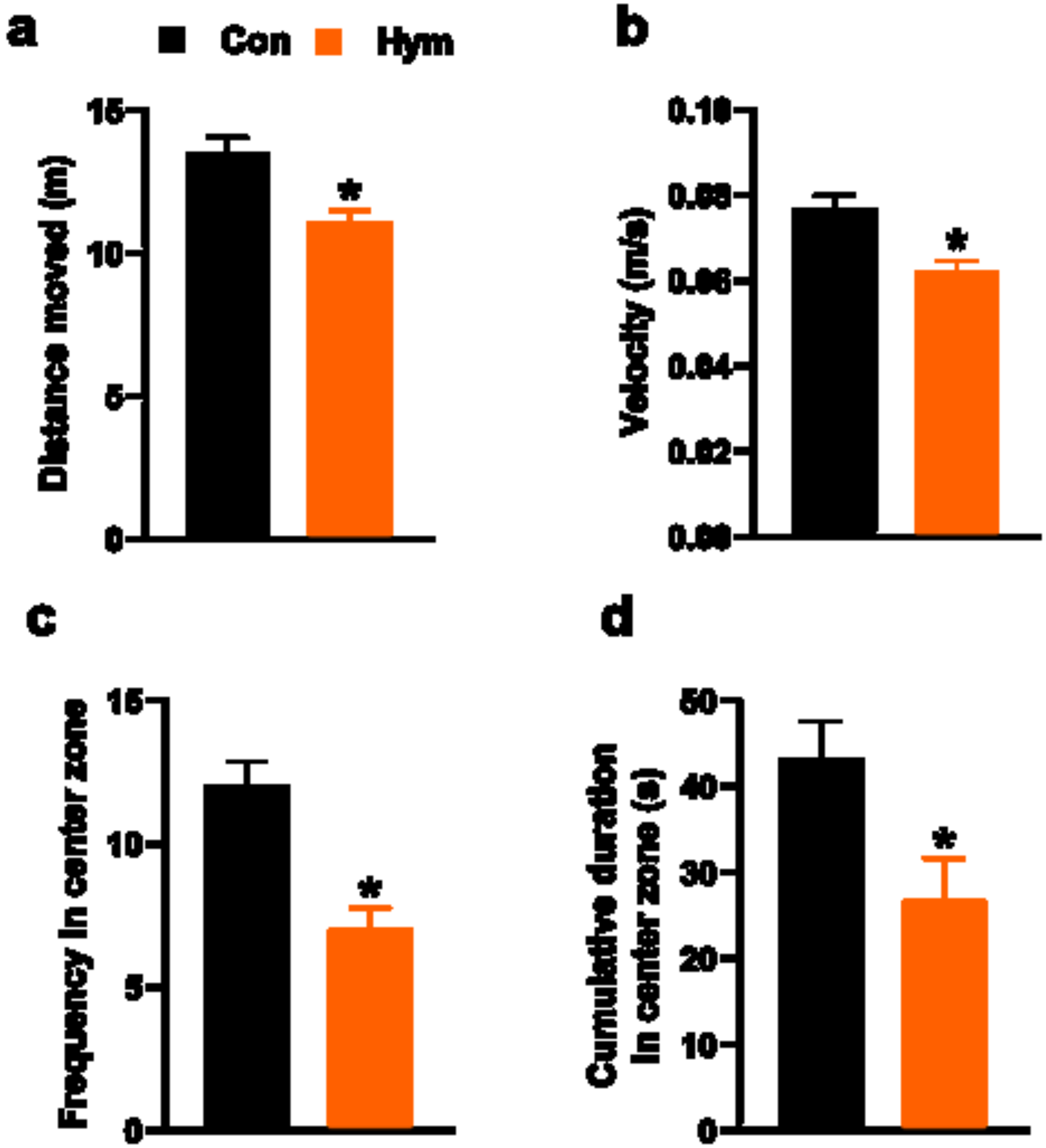

Figure 2

Click here to access/download;Figure;Fig2.tif

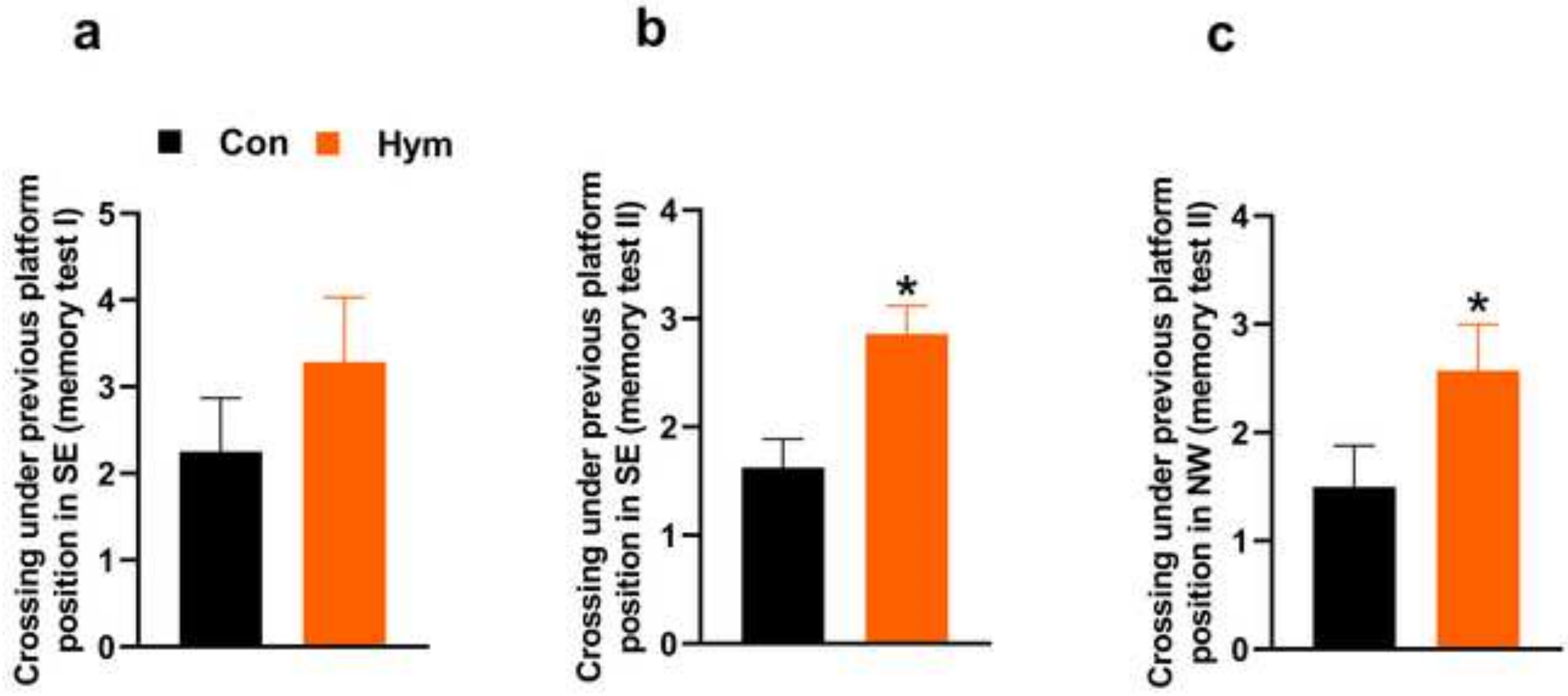

Figure 3 Click here to access/download;Figure;Fig3.tif

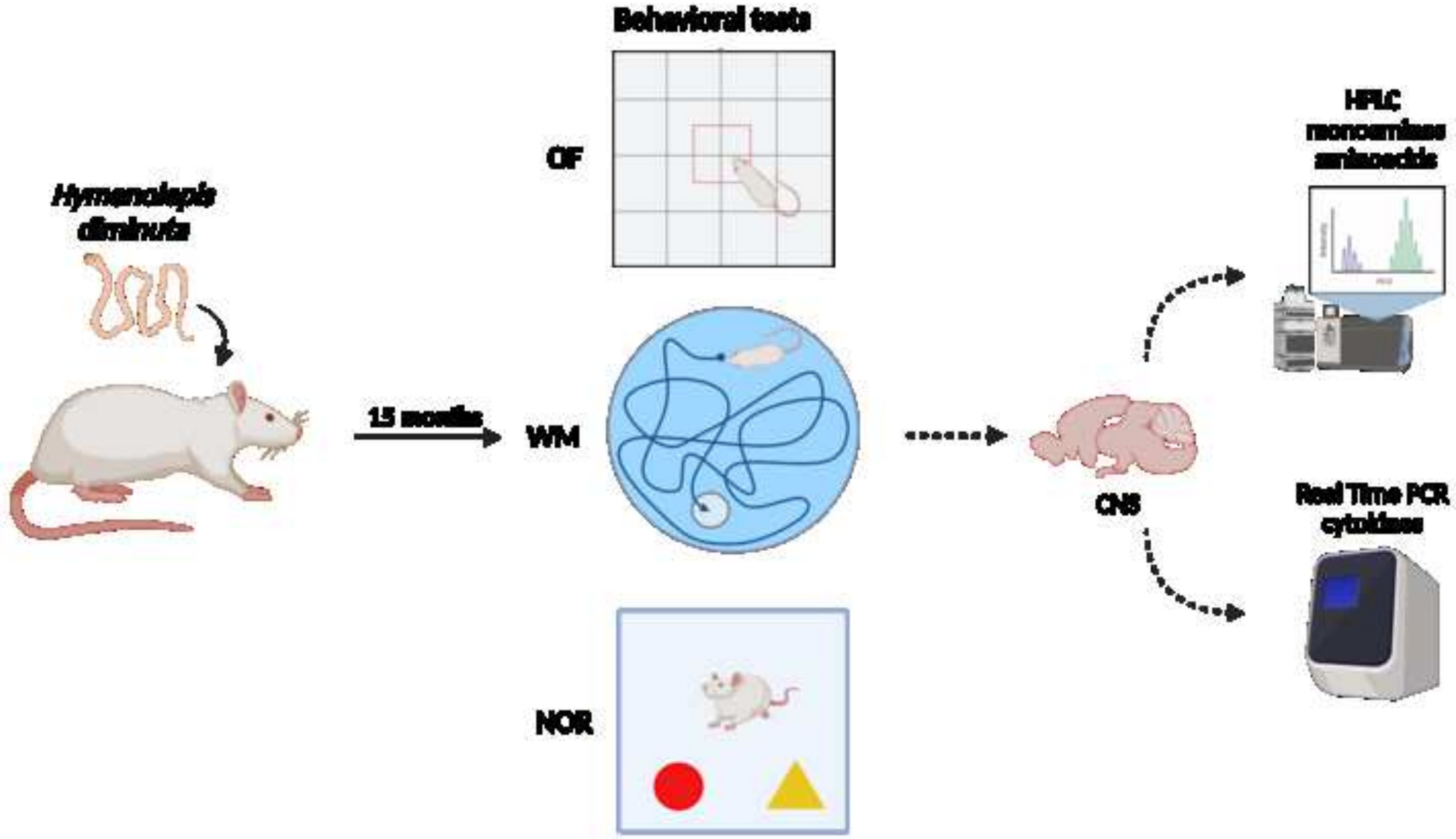

Click here to access/download;Figure;Fingerprint - graphical abstract.tif

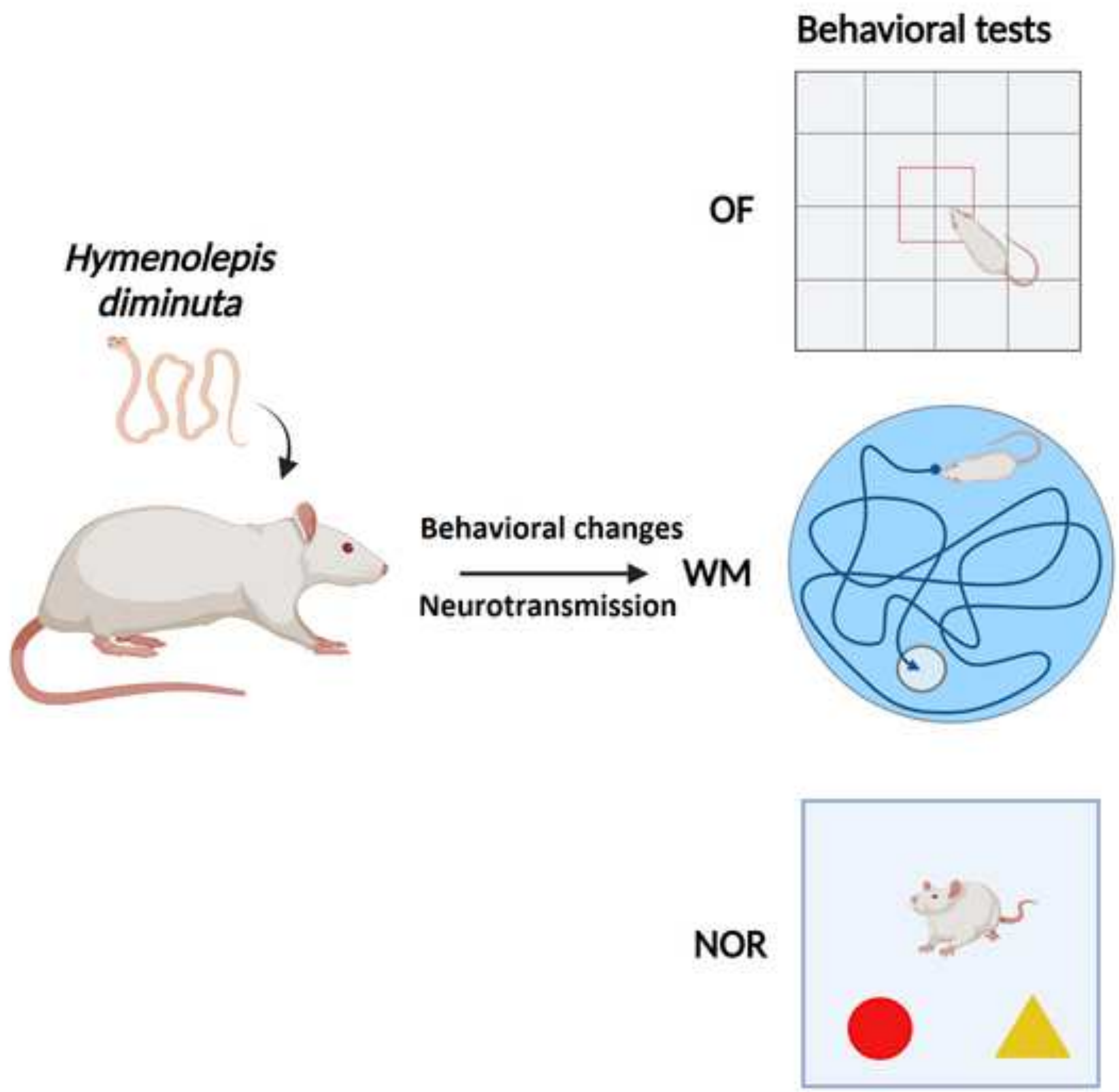

Supplement: Attachment — Submitted filename: PPATHOGENS-D-21-02092 (1).pdf [file ppat.1010330.s001.pdf]
