# Supplementary material for: Infection with intestinal helminth (Hymenolepis diminuta) impacts exploratory behavior and cognitive processes in rats by changing the central level of neurotransmitters
Source: PLoS Pathog. 2022 Mar 14;18(3):e1010330. doi: 10.1371/journal.ppat.1010330 (PMC8947016; doi:10.1371/journal.ppat.1010330)
Supplement: Attachment — Submitted filename: COVER LETTER -respons to Reviewers.docx [file ppat.1010330.s002.docx]

Prof. Dagmara Mirowska-Guzel

Department of Experimental and Clinical Pharmacology,

Medical University of Warsaw,

Centre for Preclinical Research and Technology CePT

Banacha 1B, 02-097 Warsaw, Poland

tel./fax. +48 22 116 6202

E-mail address: dagmara.mirowska-guzel@wum.edu.pl

Dear Editors,

Thank You for allowing us to respond to the Reviewer’s comments on our manuscript entitled "Infection with intestinal helminth (*Hymenolepis diminuta*) impacts exploratory behavior and cognitive processes in rats by decreasing noradrenergic and serotonergic neurotransmission" (PPATHOGENS-D-21-02092). We have revised our manuscript to incorporated the comments of the Reviewer’s as follows:

Reviewer I

\# In line with Reviewer suggestion we have added more detailed information and citation about the relationship between neurotransmitters levels and cytokines production in the brain to the Introduction. We also developed the topic of downregulation of neurotransmitters and cytokines production in response on intestinal parasite infection.

\# We have described the link between biochemical parameters and increasing level of anxiety in parallel with improved spatial memory and better recognition of new objects in infected rats.

\# Additionally, we explained why different parts of the brain were assessed for neurotransmitter levels and why the metabolites of noradrenaline were measured.

\# According to the Reviewer’s recommendation we use Summary section to discuss strengths, novelty and significance as well as general execution and scholarship.

\# According to the Reviewer’s suggestion we revised presentation of experimental data. We improved description of results, legend and supplemented the text with proper reference to the parts of the graph that were analyzed. Analysis and conclusion of molecular results was also improved.

\# According to the comments, some general knowledge included in discussion was removed or transferred to Introduction.

Reviewer II

\# According to the Reviewer’s comments Introduction has been rewritten and the tittle was changed as follows:

INFECTION WITH INTESTINAL HELMINTH (HYMENOLEPIS DIMINUTA) IMPACTS EXPLORATORY BEHAVIOR AND COGNITIVE PROCESSES IN RATS BY CHANGING THE CENTRAL LEVEL OF NEUROTRANSMITTERS.

\# Referring to the comments of the Reviewer 2 we decided to improve data description and we added additional graphs and tables illustrating the results obtained and we put an appropriate explanation in the text. We reported more data graphically or in a table to make the results more readable. In the results section, we have completed and improved graphical presentation of Water Maze data and indicated the meaning of each phase of the Morris Water Maze tests. Additionally we have defined the meaning of the indices mentioned for the novel object recognition test.

In the HPLC results we have spelled out the metabolite names at first mention, and we have explained the way of measuring neurotransmitters turn over (ratio of the metabolite vs neurotransmitter). Unfortunately, in some cases, when we measured frequency of some behavior we were unable to list the units in the Results section. The word “frequency” was not clearly used. We changed it to “number” of enumerated behaviors and hope that now it is easier to understand.

\# We agree that measurement of neurotransmitters by microdialysis is better options than HPLC post mortem because could provide a time course of changes in brain metabolites. Limitation is also lack of assessment of biochemical changes in specific brain nuclei. We plan to develop research in this direction but for the purposes of the presented manuscript it is impossible.

\# We agree that Open Field alone is insufficient to determine anxiety and in the discussion we considered it as limitations.

\# In this study we don’t analyze behavior the same animal before and during early and late infection and this limitation we have included in Discussion.

\# We can’t change numeration of Figure 3, because the numbering of tables and figures results from the order in which they appear in the text (Methods section is at the end of the manuscript).

\# According to the suggestions we delete the word “attitude” and change “activities” to “experiments”. The phrase “reduced the discomfort of the animals caused by the additional procedure” has been omitted.

\# In Methods we have indicated when and how grooming, climbing, urination, and defecation were assessed. We have indicated the EthoVision settings for movement detection in the methods. In Methods, we have listed the brain tissue dissected, and explained how they were frozen.

\# We improved the Statistics section.

\# We defined more clearly the limitations of our findings in the discussion. We have discussed the possibility that some of the behavioral deficits could be associated with malaise, fatigue/nutritional deficits associated with infection, and may not relate strictly to anxiety or an altered emotionality.

In the discussion some parts has been rewritten or removed.

\# We added citations about influence of parasites on serotonergic activity and we have discussed meaning of alteration of IL-6 in the hippocampus and its effects on learning and memory.

\# In our experiment we used males because females are less stable in terms of behavior due to fluctuations in the level of sex hormones. Therefore, in behavioral studies, the usage of females, which often require cycle synchronization, is more challenging. Further studies comparing the effect of tapeworm infection by gender may be planned.

#. There were not significant differences in the rat body weight between the infected and uninfected rats. Along the infection period the body weight of infected and uninfected animals was ranging between:.

|  | 6 month p.i. | | 12 months p.i | | 15 month p.i. | | 15 month p.i. | |
| --- | --- | --- | --- | --- | --- | --- | --- | --- |
|  | min | max | min | max | min | max | min | max |
| uninfected | 375 | 397 | 410 | 460 | 318 | 565 | 297 | 509 |
| infected | 368 | 395 | 415 | 485 | 465 | 509 | 430 | 503 |

\# According to the Reviewer’s suggestion we provide more specific information about *H. diminuta’s* life cycle (how does it get into the host) and what is known about how it changes the microbiome and immune system as well as how might those changes alter immune or brain function. This issue is added to the improved version of the Discussion based on the available literature data, however, we also plan to investigate this correlation in the future studies.

\# We provided citations for the behavioral assays and infection methodology. The *H. diminuta* tapeworm has been breeding in our department for over forty years. A number of studies have been published and our experience shows that the administration of an invasive dose in the amount of 5-6 cysticercoids is optimal. Infection did not affect the condition and mortality of the animals, which is confirmed by the available literature data.

\# We explained more clearly procedure of animals’ infection.

Six-week-old *H. diminuta* cysticercoids were extracted from dissected *Tenebrio molitor* beetles under a microscope (magnification 100 x) and used to infect three-month-old rats by oral uptake of six cysticercoids of *H. diminuta* suspended in a 0.6% sterile saline solution per rat. To verify the presence of adult parasites, fecal samples were taken from the animals and examined for the presence of tapeworm eggs under a microscope (magnification 400 x) along the experiment period. We did not observed the higher body temperature in infected rats. Previously published data indicate the relatively slight immune response to the infection (Goswami 2011, Williamson 2016) with no significant influence on rat mortality, body weight and condition. Research by Goswami et al. (2011) showed that Wistar rats infected with *H. diminuta* showed reduced hemoglobin values (HGB), while other parameters, including the number of red blood cells (RBC), erythrocyte sedimentation rate (ESR), hematocrit (Hct), white blood cell count (WBC) and differential leukocyte count (DLC) were not significantly changed. We agree that more detailed data collected in different infection periods are important and these will be taken into account in our future detailed studies.

\# Correlation between the microbiome, immune response and rat neurobiology is now provided and discussed in the Discussion. We added data related to the influence of *H. diminuta* on rat microbiome and behavior. We also discussed in more details pioneering research by Williamson et al (2016), who studied interactions between tapeworm infection, the microbiome, and the neurobiology and behavior of infected rats.

\# The aim of our study was to check whether a chronic infection would have an effect on the behavior and levels of neurotransmitters in rats. The results of our research indicate statistically significant differences in the level of neurotransmitters that affect the behavior of animals in various behavioral tests. All your other valuable suggestions and comments will be taken into account when planning further research and applying for funding. We are very grateful for pointing them out In future studies, we are going to explain how this happens and what factors are affected by the infection. Animals of different ages and at different stages of infection will be examined. Factors such as systemic immune response, microbiome, local response, metabolome etc. will be taken into account. It should be taken into account that the parasite burden and the age of the infected rats may also play a role. Nevertheless, our research clearly shows differences between infected and non-infected animals. Appropriate statements are added into the Discussion. We believe that the role of parasites infection on CNS functioning is still a problem that has not been adequately presented and explained. There are only a few articles that take up this topic. In this respect our study is innovative and elucidates some new aspects of the behavioral and biochemical changes response to the parasite infection. In this study we discovered that parasite infection lowered the level of neurotransmitters and proinflammatory factors which are reflected in changes of animal behavior.

I hope that we have revised the manuscript to Your satisfaction, and that You will reconsider our study for publication in PLOS Pathogens. Please do not hesitate to contact me in case of having any further comments or questions.

Sincerely Yours,

Dagmara Mirowska-Guzel

Daniel Młocicki
